# Crystal structure of Psb27 from *Arabidopsis thaliana* determined at a resolution of 1.85 Å

**DOI:** 10.1007/s11120-017-0450-3

**Published:** 2017-11-02

**Authors:** Cheng Xingxing, Liu Jiuyang, Zhang Huan, Li Fudong, Zhang Shuya, Xu Min, Ruan Ke, Wang Yuhua, Fu Aigen

**Affiliations:** 1 Chinese Education Ministry’s Key Laboratory of Western Resources and Modern Biotechnology, Key Laboratory of Biotechnology Shaanxi Province, College of Life Sciences, Northwest University, 229 North Taibai Road, Xi’an, 710069 Shaanxi China; 2 Hefei National Laboratory for Physical Science at the Microscale, School of Life Sciences, University of Science and Technology of China, 96 Jinzai Road, Hefei, 230027 Anhui China

**Keywords:** Photosystem II, Assembly and repair, Psb27, Arabidopsis, Crystal structure

## Abstract

Proper biogenesis and maintenance of photosynthetic thylakoid membrane complexes are essential for the photosynthetic light reactions. A thylakoid lumenal protein, Psb27, plays a vital role in assembly or/and maintenance of photosystem II (PSII). In cyanobacteria, it is a small lipoprotein docked to the lumenal side of PSII, and functions in the assembly of the Mn_4_Ca cluster and in the PSII repair cycle. However, Psb27 from *Arabidopsis thaliana* is not a lipoprotein, and it is involved in PSII repair and acclimation to fluctuating light stress, suggesting a functional divergence between Arabidopsis Psb27 and cyanobacterial Psb27s. To gain a better understanding of Psb27 from higher plants, we determined the crystal structure of Arabidopsis Psb27 by X-ray crystallography at a resolution of 1.85 Å. The structure of Arabidopsis Psb27 is a four-helix bundle, similar to its orthologues from cyanobacteria. However, there are several structural differences between Arabidopsis Psb27 and cyanobacterial Psb27s concerning the overall molecular shape, the N- and C-terminal structures, and the surface charge. These differences suggest that Psb27 from higher plants and cyanobacteria may function differently.

**Electronic supplementary material:**

The online version of this article (doi:10.1007/s11120-017-0450-3) contains supplementary material, which is available to authorized users.

## Introduction

Photosystem II (PSII) is the electron donor of the photosynthetic electron transfer chain, where it catalyzes the light-driven oxidation of water and the reduction of plastoquinone (Nelson and Yocum 2006; Barber 2006). The structures of PSII from cyanobacteria (Ferreira et al. 2004; Loll et al. 2005; Umena et al. 2011), a red algae (Ago et al. 2016), and spinach (Wei et al. 2016) determined by X-ray crystallography or NMR revealed that at least 20 protein subunits, numerous cofactors, and pigment molecules reside in a functional PSII complex.

The de novo assembly or biogenesis of PSII is a highly coordinated and complex process (Komenda et al. 2012a; Lu 2016). It occurs similarly in cyanobacteria and higher plants starting with the formation of the PSII core (including D1, D2, PsbE and F), followed by the assembly of the PSII monomeric reaction core complex after the addition of CP43 and CP47. The functional PSII monomer is obtained by incorporating low molecular mass subunits and the oxygen evolving complex, and finally the dimeric PSII is built up (Aro et al. 2005; Nixon et al. 2010). PSII is prone to light-induced damage, and the D1 subunit is the primary target for photo-damage. In order to maintain PSII homeostasis, photosynthetic organisms have developed a mechanism known as the “PSII repair cycle,” in which damaged D1 is replaced by a newly synthesized copy and then the PSII complex is reassembled and reactivated again (Adir et al. 2003). The PSII repair cycle and the biogenesis of PSII are two different processes, but they share common components (Järvi et al. 2015). A large number of auxiliary proteins, including Psb27, have been shown to be involved in the assembly and/or the repair cycle of PSII (Nixon et al. 2010; Lu 2016).

Psb27 was originally identified as a component of an active PSII complex isolated from *Synechocystis* sp. PCC 6803 (Kashino et al. 2002a) and *Thermosynechococcus vulcanus* (Kashino et al. 2002b). However, further experiments revealed that it is not present in the crystal structures of cyanobacterial PSII (Ferreira et al. 2004; Loll et al. 2005; Umena et al. 2011). Phylogenetic studies suggested that Psb27 exists in all oxygenic photosynthetic organisms except *Gloeobacter violaceus* (Gupta 2009). In most cyanobacteria, Psb27 is a thylakoid lumenal lipoprotein attached to the thylakoid membrane through its lipid-modified N-terminus (Nowaczyk et al. 2006; Fagerlund and Eaton-Rye 2011).

The Psb27-containing PSII assembly intermediate (PSII-Psb27) isolated from *T. elongatus* is devoid of PsbO, V, and U, suggesting that Psb27 plays a role in the assembly of the Mn_4_Ca cluster of the oxygen evolving complex (Nowaczyk et al. 2006; Mamedov et al. 2007). The involvement of Psb27 in Mn_4_Ca cluster assembly was also found in *Synechocystis* sp. PCC 6803 (Roose and Pakrasi 2008). However, the PSII-Psb27 complex isolated from *Synechocystis* sp. PCC6803 still contains considerable amounts of PsbO, but not PsbV or PsbU (Liu et al. 2011a), indicating that the isolation of PSII-Psb27 complex is species- or preparation-dependent. In addition to its role in PSII assembly, Psb27 was found to play a role in PSII repair as well (Grasse et al. 2011).

Several approaches have been attempted to map the Psb27 binding site on PSII to uncover the mechanism of Psb27 action in cyanobacteria. A Psb27 structure determined by NMR and in silico docking experiments suggested that the binding site of Psb27 and PsbO is partially over-lapped in proximity to CP47 (Cormann et al. 2009). However, another NMR experiment and an X-ray crystallography study indicated that Psb27 likely binds to a site next to CP43 on PSII (Mabbitt et al. 2009; Michoux et al. 2012). The physical interaction between Psb27 and CP43 was confirmed by cross-linking experiments (Liu et al. 2011b; Komenda et al. 2012b). Further detailed analysis showed that Psb27 interacts with lumenal loops of CP43 (Liu et al. 2013; Cormann et al. 2016).

So far, little is known about how Psb27 functions in higher plants. There are two Psb27 isoforms in *Arabidopsis thaliana*, AtPsb27 (At1g03600) and AtLPA19/Psb27-H2 (At1g05385) (Chen et al. 2006; Wei et al. 2010). Phylogentically, AtPsb27 is more closely related to cynobacterial Psb27 than AtLPA19, and both AtPsb27 and AtLPA19 display a low sequence identity to their cyanobacterial homologs (Chen et al. 2006; Wei et al. 2010). The two Psb27 isoforms from higher plants do not contain lipobox sequences and N-terminal cysteines, and they are not membrane-anchored lipoproteins (Chen et al. 2006; Wei et al. 2010; Hou et al. 2015). The stunted growth phenotype of *atlpa19* mutant plants and the interaction between AtLPA19 and the C-terminal region of D1 suggested that AtLPA19 plays an important role in the D1 precursor processing (Wei et al. 2010). An Arabidopsis mutant lacking AtPsb27 was deficient in recovery of PSII activity after photoinhibition, suggesting that AtPsb27 is required for the efficient repair of photodamaged PSII (Chen et al. 2006). Another study suggested that AtPsb27 could play a role in state transitions to balance the energy distribution between PSII and PSI (Dietzel et al. 2011). The Arabidopsis *atpsb27* mutant is highly sensitive to a fluctuating light, implying that AtPsb27 is an important factor for plants in response to a varying light condition (Hou et al. 2015).

The differences observed for the physiological roles played by Arabidopsis Psb27 homologs and cyanobacterial Psb27s raise the question whether Psb27s from cyanobacteria and higher plants work in the same way. In an attempt to shed light on this question and to gain a better understanding of Psb27 from higher plants, we solved the crystal structure of AtPsb27 by an X-ray diffraction at a resolution of 1.85 Å. In spite of the low sequence identity of the protein, Psb27 from Arabidopsis forms a 4-helix bundle structure, similar to the structures of Psb27 from *Synechocystis* sp. PCC6803 (Cormann et al. 2009, 2KND; Mabbitt et al. 2009, 2KMF) and *T. elongatus* (Michoux et al. 2012, 2Y6X). However, the crystal structure reveals major differences between AtPsb27 and cyanobacterial Psb27, with respect to the global molecular shape, the N- and C-terminal structures, and the surface charge. Our study suggests that Psb27 proteins from higher plants and cyanobacteria may be involved in the assembly and repair of PSII through different mechanisms.

## Results and discussions

### Expression of the AtPsb27 protein

**Fig. 1:**
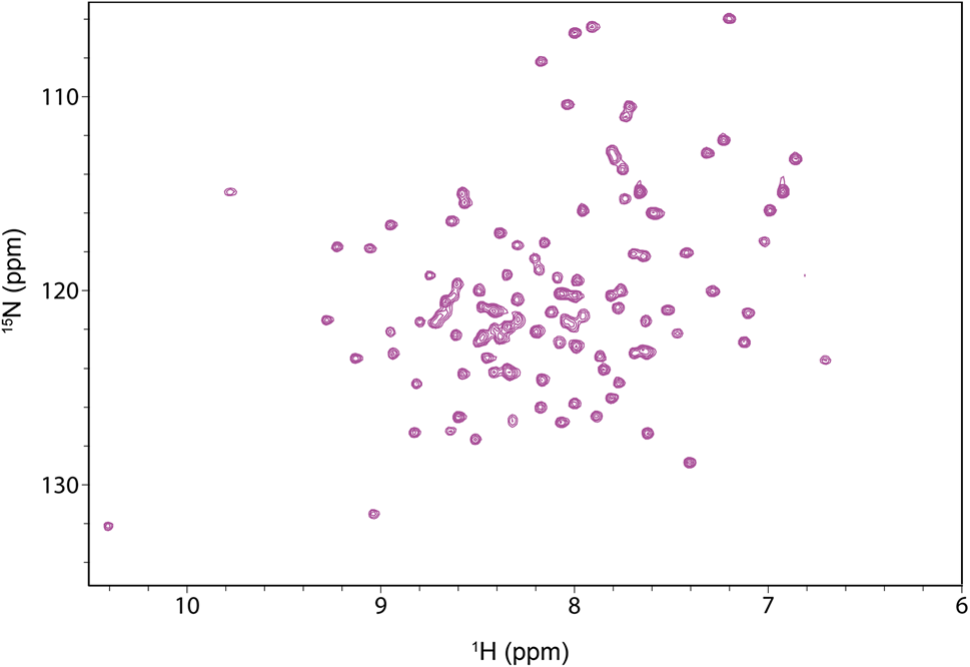
HSQC spectrum of AtPsb27 at pH 6.7. The ^15^N-labeled AtPsb27 was concentrated to 0.2 mM, and the HSQC spectrum was recorded in a buffer containing 20 mM sodium phosphate, 250 mM NaCl, 2.5 m M DTT, 2.5 mM Na_2_EDTA, and 0.002% NaN_3_ at pH 6.7

The full length protein of Psb27 from *A. thaliana* is composed of 174 amino acids (AA), with a 68 AA-long chloroplast targeting peptide at the N-terminus. To determine the structure of Psb27 from *A. thaliana*, its mature form (AtPsb27) was successfully over-expressed as a GST-AtPsb27 fusion protein in *E. coli*. The recombinant protein was isolated and purified, and treated with TEV protease to remove the GST tag and generate the AtPsb27 protein (Fig. 1S). The non-tagged AtPsb27 protein was further purified by size exclusion chromatograph with a superdex-75 column, and then the AtPsb27 protein sample was subjected to heteronuclear single quantum coherence (HSQC) spectrum analysis and crystallization.

### ^1^H–^15^N HSQC spectrum of AtPsb27

Before determining the AtPsb27 structure, we examined the protein aggregation and folding status. TheΔpH across the thylakoid membrane is estimated to be 3–3.5, and the pH value in the thylakoid lumen space would range from 5.5 to 7.8 when photosynthesis operates under optimum conditions (Takizawa et al. 2007; Tikhonov et al. 2008). Therefore, we first analyzed the solubility of AtPsb27 over a range of pH values of 4.5, 5.5, 6.7, 7.0, and 7.5, respectively. We found that the AtPsb27 protein aggregates at a pH below 5.5, suggesting that AtPsb27 is not stable under low pH conditions. It was reported that Psb27 from *Synechocystis* sp. PCC6803 remains stable under a range of pH values from 3.5 to 9.0, and pH variations have little effect on its conformation (Mabbitt et al. 2009). It may not be surprising that AtPsb27 is sensitive to lower pH conditions, considering that AtPsb27 is a heavily positively charged protein with a pI of 9.22. In contrast, Psb27 from *Synechocystis* sp. PCC6803 is stable in lower pH probably due to its more neutral status with a pI of 8.05.

Because the AtPsb27 protein is stable at pH 6.7, 7.0, and 7.5, we further subjected ^15^N radio-labeled AtPsb27 samples to heteronuclear single quantum coherence (HSQC) spectrum analysis. The HSQC spectra of AtPab27 were almost identical at pH 6.7, 7.0, and 7.5. We therefore only present the spectrum at pH 6.7 in this paper (Fig. 1). The HSQC spectrum showed that the resonance signals were well dispersed, indicating the protein was folded in a proper way.

### Crystal structure of AtPsb27

**Fig. 2:**
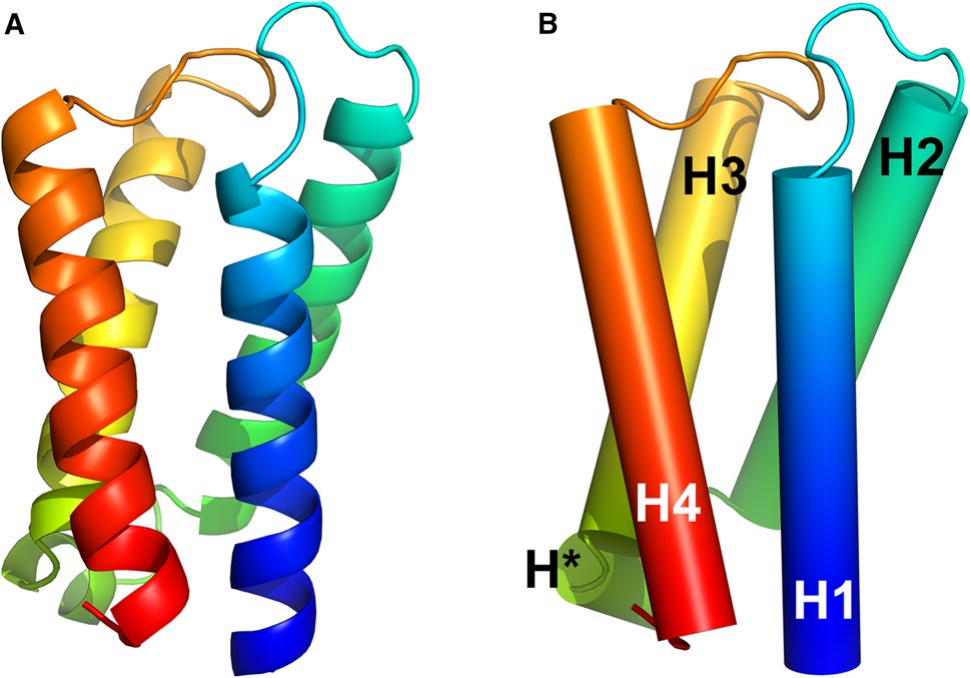
Crystal structure of AtPsb27 (PDB 5 × 56). **a** Ribbon model, **b** backbone model. The structure was obtained by molecular replacement and visualized using PyMol. Alpha helices are indicated as H1–H4 and H*. Both models are colored in rainbow (from blue N-terminus to red C-terminus)

AtPsb27 crystalized at a concentration of 22 mg/ml in 2.0M potassium/sodium phosphate solution at pH 7.0, and the crystals of AtPsb27 were observed after 3 days (Fig. 2S). The long needle-shaped appearance of AtPsb27 crystals is different from crystals of Psb27 from *T. elongatus* (TePsb27, 2Y6X), which displayed a cubic shape (Michoux et al. 2012). The 3-D crystal structure datasets of AtPsb27 were acquired at a resolution of 1.85 Å, and a molecular fine replacement was attempted using the structure of TePsb27 (Michoux et al. 2012, 2Y6X) as a search model. Details of data collection and refinement statistics are presented in Table 1.

**Table 1:** X-ray crystal data collection and refinement statistics of AtPsb27

PDB ID	5 × 56
Data collection	
Space group	C 1 2 1
Cell dimensions	
a, b, c (Å)	86.26, 62.40, 38.96
α, β, γ (°)	90.00, 112.63, 90.00
Wavelength(Å)	0.979
Resolution (Å)	39.81–1.85 (1.95–1.85)*
Completeness (%)	98.8(98.5)
Redundancy	4.9(4.9)
*R*_sym_ or *R*_merge_ (%)	7.9(57.0)
*I*/σ*I*	11.6(2.9)
Refinement	
No. reflections used/free	16,080/752
*R*_work_/*R*_free_	21.3/26.1
R.m.s. deviations	
Bondslengths (Å)	0.007
Bond angles (°)	0.796
*B*-factors (Å^2^)	
Protein	31.55
Water	32.01
No. atoms	
Protein	1595
Water	49
Ramachandran plot	
Favored/allowed/outlier(%)	99.5/0.5/0.0

*Values in parentheses are for highest-resolution shell

**Fig. 3:**
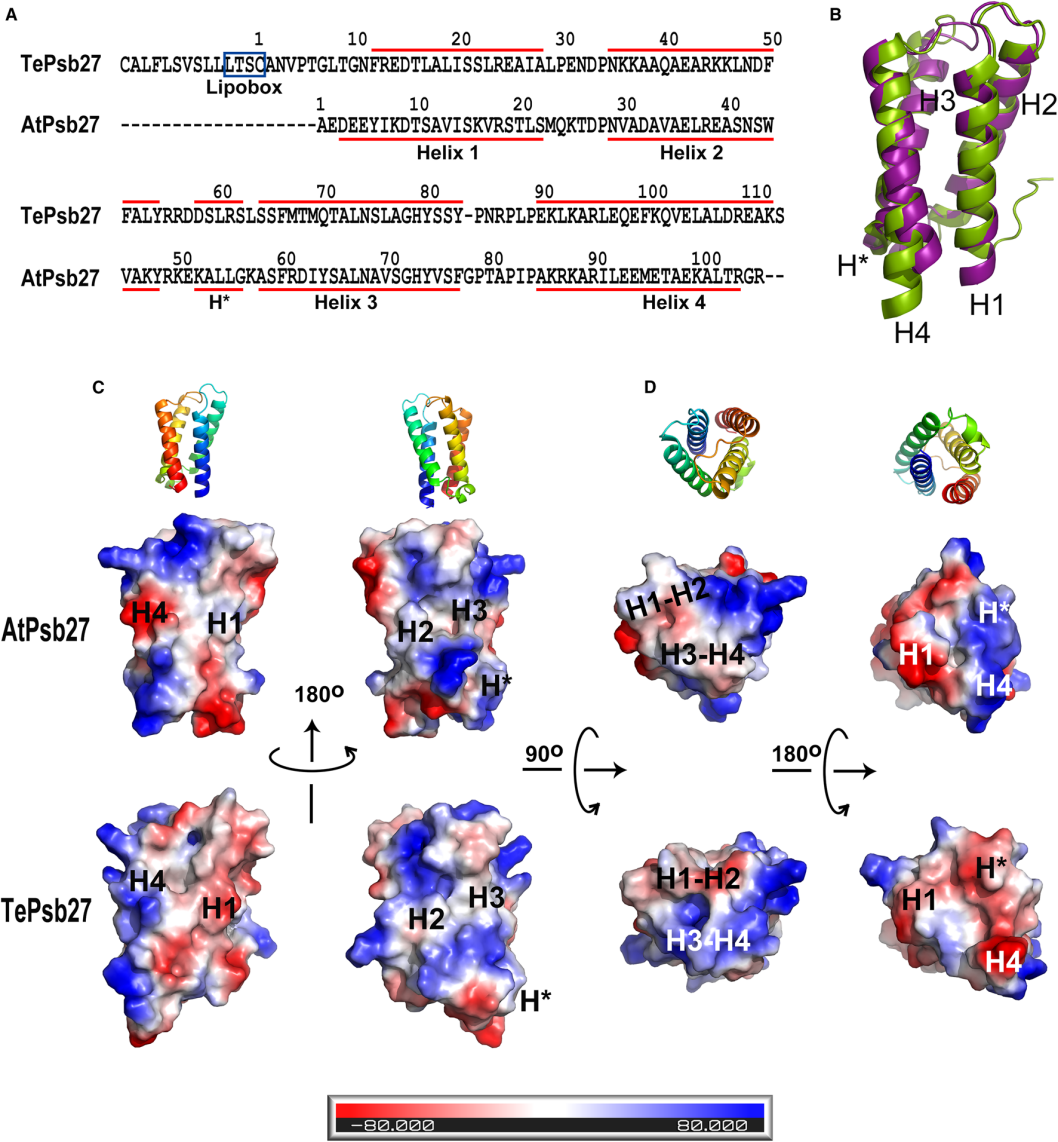
Comparison of AtPsb27 and TePsb27. **a** Sequence alignment of AtPsb27 and TePsb27. The mature sequence of AtPsb27 and the full length sequence of TePsb27 were aligned with ClustalW2 program. The lipobox in the full length TePsb27 is boxed with blue line. The α-helices of Psb27 are underlined in red, and residues are numbered according to the mature sequences. **b** Alignment of the backbone structures of AtPsb27 (magenta) and TePsb27 (green) in the ribbon model. **c, d** Comparison of surface charge distributions between AtPsb27 and TePsb27. The range of surface charge is shown from − 80 kT/e (red) to + 80 kT/e (blue).The corresponding ribbon models of AtPsb27 are shown at the top. **c** Front view (left) and back view (right). **d** Top view (left) and bottom view (right)

The overall 3-D structure of AtPsb27 (5 × 56) is similar to the three structures obtained from cyanobacterial Psb27 proteins (Cormann et al. 2009, 2KND, 4.1 Å; Mabbitt et al. 2009, 2KMF, 1.8 Å; Michoux et al. 2012, 2Y6X, 1.6 Å). The AtPsb27 structure reveals as a helix bundle with a relatively flat top and bottom, consisting of four long alpha helices (H1–H4) as well as a small helix between H2 and H3, designated as H* (Fig. 2). The four alpha helices, H1–H4, span residues 3–22, 30–49, 58–76, and 85–104, respectively, and are arranged in an up-down-up-down anti-parallel fold to form the bundle body (Figs. 2, 3a). The loops H1–H2 and H3–H4 are located on the top of the structure. The well-conserved P-Φ-P motif (where Φ is I, L, or V) resides in the loop H3–H4, and is exposed to the AtPsb27 surface (Fig. 2). The small helix H* is formed by residues 52–56, and constitutes the bottom area of the AtPsb27 structure (Fig. 2).

### Comparison between structures of AtPsb27 and TePsb27

Psb27s in higher plants are highly conserved in their amino acid sequences (Fig. 3S), suggesting that they have similar tertiary structure. Moreover, the structures of three cyanobacterial Psb27 proteins are almost identical (Cormann et al. 2009; Mabbitt et al. 2009; Michoux et al. 2012). Therefore, we attempted to uncover the structural differences between Psb27 proteins from higher plants and cyanobacteria by comparing the structure of AtPsb27 (5 × 56) to the structure of Psb27 from *T. elongatus* (TePsb27, 2Y6X) (Fig. 3).

Overall, AtPsb27 bears a sequence identity of 34% and a sequence similarity of 57% to TePsb27 (Fig. 3a); their tertiary structures are mostly similar to each other, both forming 4-helix bundle structures, but some structural differences are clearly observed between these two structures (Fig. 3b–d). Firstly, H1 and H4 are positioned more closely in AtPsb27 than they are in TePsb27, which makes the AtPsb27 bundle tighter than in TePsb27 (Fig. 3b). Secondly, the length of the bundle-forming alpha helices (H1–H4) is almost the same in AtPsb27, each with 20 residues except H3 with 19 residues. Therefore, AtPsb27 folds into a typical bundle structure with relatively flat top and bottom surfaces (Fig. 3b, c). However, H4 of TePsb27 is six residues longer than H1 (23 residues vs. 17 residues). As a result TePsb27 could not form an even bottom; instead it folds into a more irregular shape with a protruding bottom (Fig. 3b, c). Thirdly, on the top surface of AtPsb27, the loop H3–H4 folds toward to the loop H1–H2, resulting in a very small space between these two loops. Compared to AtPsb27, the loop H3–H4 of TePsb27 is one residue shorter, which leads to a larger distance between the H3–H4 and H1–H2 loops. Therefore, there is a visible pocket structure at the top of TePsb27, but not at the top of AtPsb27 (Fig. 3b, d). In addition, there are also differences between the N-terminal extension of AtPsb27 and TePsb27. Similar to other cyanobacterial Psb27 proteins, TePsb27 is a lipoprotein, containing a long flexible N-terminal fragment of 11 residues. In contrast, the N-terminal extension of AtPsb27 is very short, and composed of only two residues. (Fig. 3a, b, d).

### Surface charge differences between AtPsb27 and TePsb27

Another major difference between AtPsb27 and TePsb27 comes from the charges present on the protein surfaces.

At first, the N-terminal tail of TePsb27 is nearly neutral and contains two threonine residues and two asparagine residues, which could promote the interaction of TePsb27 with the phosphate groups of the thylakoid membrane (Fig. 3a, c). In contrast, the N-terminal region of AtPsb27 consists of a stretch of negatively charged residues (Fig. 3a, c), which makes it unlikely for AtPsb27 to bind the membrane through its N terminus.

Secondly, the electrostatic charges on the bundle-forming alpha helices are very different between these two structures, except for H3 which is nearly neutral in both proteins (Fig. 3a, c). More specifically, H1 of AtPsb27 is slightly positively charged due to the K8, K16, and R18 residues, whereas the surface of H1 in TePsb27 is covered with negative charges because of E15, D16, and E25 (Fig. 3a, c). In the case of H2 of AtPsb27, its N terminal domain contains negative charges and its C-terminal half is strongly positively charged, whereas H2 of TePsb27 is almost fully positively charged because of two K residues in the N-terminus and two K residues in the middle (Fig. 3a, c). H4 of AtPsb27 is composed of two positively charged ends and a strongly negatively charged central region due to the short EEME stretch (residues: 93–96), while H4 of TePsb27 is highly positively charged with a negatively charged N-terminal tip because of E110 (Fig. 3a, c).

In addition, the surface charges of both the top and bottom regions are also different between AtPsb27 and TePsb27 (Fig. 3c, d). In AtPsb27, the top surface is almost neutral with a small positively charged patch contributed by K25 in loop H1–H2, while in TePsb27, the negatively charged loop H1–H2 and the slightly positively charged loop H3–H4 form a ring-shaped pocket, which is highly positively charged inside (Fig. 3c, d). The bottom of AtPsb27 is half positively charged and half negatively charged. The positive area consists of its H* and its C terminus, and the negative area is mainly contributed by the N terminus of the protein (Fig. 3c, d). However, all components constituting the bottom of TePsb27 are basically negatively charged, including H*, the N-terminal region of H1, and the C-terminus of TePsb27, which makes the TePsb27 bottom area mostly negatively charged (Fig. 3c, d).

## Summary

In this study, we resolved the crystal structure of AtPsb27 at a resolution of 1.85 Å, which is the first Psb27 structure from higher plants. AtPsb27 shares 34% sequence identity and 55% similarity to TePsb27 and it is not a lipoprotein like TePsb27. The N- and C-terminal regions of AtPsb27 are shorter than those of TePsb27. As to the 3-D structure, AtPsb27 is a regular bundle with even top and bottom surfaces, while TePsb27 looks more irregular due to the different lengths of its four body-forming helices. The electrostatic charges present on the surface of AtPsb27 and TePsb27 are different from almost every point of view. Based on the comparison of AtPsb27 and TePsb27, it is evident that they are two distinct proteins with their own unique features. The remarkable structural differences between AtPsb27 and TePsb27 imply that they may interact with different proteins and may function through different mechanisms. AtLPA19, the second Psb27 homolog in Arabidopsis, bears a very low sequence identity and similarity to AtPsb27 and TePsb27 (Fig. 4S), and it is likely that the structure of AtLPA21 differs from those of AtPsb27 and TePsb27 in many aspects too.

Psb27 and LPA19 are distantly related to each other, but both are highly conserved in their own evolutionary linage (Wei et al. 2010). Due to the high conservation of Psb27 from higher plants, the structural differences between AtPsb27 and TePsb27 likely reflect functional differences between higher plants and cyanobacteria. These differences suggest that Psb27 of higher plants might have evolved new functions from its prokaryotic ancestor, in order to adapt to environmental changes which occurs during evolution from an aquatic to a land environment. It is worthy to point out that there are about 10 conserved charged residues amongst Psb27 proteins from higher plants, which are not found in cyanobacteria, indicating that they might be related to the physiological functions of Psb27 in higher plants. Further research should be focused on those residues, to address the question how Psb27 is involved in the process of sensing environmental conditions, and adjusts the photosynthetic electron transfer chain accordingly.

## Materials and methods

### Plant materials and growth conditions

*Arabidopsis thaliana* seeds of columbia-0 ecotype were sown on soil and cold-treated for 2 days, and then grown under a long day condition (16-h light and 8-h dark cycle) at a temperature of 23 °C, and an illumination of 70–100 µmol/m^2^/s.

### DNA extraction

Genomic DNA was extracted from 2-week-old Arabidopsis seedlings with a DNA extraction kit (TIANGEN, China) following the manufacturer’s instruction.

### Construction of GST-AtPsb27 fusion protein expressing vector

AtPsb27, the mature form of Psb27 from Arabidopsis, corresponds to the region of AA 69-174 of the full length protein. The coding sequence (CDS) of AtPsb27 was amplified by PCR and cloned into *Nde*I and *Xho*I sites of a GST fusion vector pGEX4T-2 (GE Healthcare, USA), directly downstream of a TEV protease cleavage site, and the resulting construct is designated as pGST-AtPsb27. Because the Arabidopsis *Psb27*gene (*At1g03600*) does not contain any intron, genomic DNA was used as the template to amplify AtPsb27’s CDS. The primers used were Psb27-NdeI-F (5′-CGCCATATGGCTGAAGATGAAGAGTAT-3′) and Psb27-XhoI-R (5′-CCGCTCGAGTCATCTTCCTCTTGTGAGAGCT-3′).

### Expression and purification of AtPsb27 protein

The plasmid pGST-AtPsb27 was transformed into the *E. coli* strain BL-21(DE3). Cells were cultured in Luria–Bertani medium (1% tryptone, 0.5% yeast extract, and 1% NaCl) at 37 °C to an OD600 of 0.8–1.0 and then induced with 3 mM isopropyl β-d-thiogalactoside at 16 °C for 20 h. After that, cells were harvested by centrifugation, and re-suspended in binding buffer (20 mM PBS, 1 M NaCl, pH 8.0, 100 µM phenylmethylsulfonyl fluoride, 10 mg/ml lysozyme). After one-hour incubation on ice, cells were disrupted by sonication, and pelleted by centrifugation. The GST-AtPsb27 recombinant protein was purified from the supernatant by affinity chromatography over glutathione-Sepharose 4B (GE Healthcare, USA). First, glutathione-Sepharose beads were incubated with the supernatant for 4 h at 4 °C to bind GST-AtPsb27, and then washed three times with washing buffer (20 mM PBS, 1M NaCl, 1% Triton X-100, pH 8.0). Finally, AtPsb27 was cleaved off from the fusion protein bound on beads by overnight digestion at 4 °C with 100 µM TEV enzyme in a buffer containing 20 mM Tris–HCl pH 8.0 and 200 mM NaCl. The protein was further purified through a Superdex-75 column by a gel filtration method.

The ^15^N-labeled AtPsb27 was induced and purified using the same procedures as described above, except that cells carrying the pGST-AtPsb27 construct were cultured in SV40 medium (20 mM K_2_HPO_4_·3H_2_O, 100 mM KH_2_PO_4_, 10 mM ^15^NH_4_Cl, 100 µM MgSO_4_, 1 ml VB_1_, 2.5 g/l glucose).

### HSQC spectrum recording

The ^15^N-labeled AtPsb27 was used for the heteronuclear single quantum coherence (HSQC) spectrum analysis followed the procedure used by Liu et al. (2017). Protein was concentrated to 0.2 mM in a buffer containing 20 mM sodium phosphate, 250 mM NaCl, 2.5 mM DTT, 2.5 mM Na_2_EDTA, and 0.002% NaN_3_ at pH6.7, 7.0, and 7.5, respectively. The HSQC spectra were acquired on an Agilent 700 MHz spectrometer equipped with a probe at 16 °C.

### X-ray crystallography and structure determination

Protein crystallization and structure determination were conducted following a protocol previously described by Liu et al. (2017). In brief, the purified AtPsb27 was dialyzed against a buffer of 20 mM Tris–HCl (pH 8.0) and 200 mM NaCl at 4 °C and then concentrated to 22 mg/ml using an Amicon Centricon YM-10 (Millipore, Billerica, MA, USA). Crystallization trials were carried out using the sitting drop vapor diffusion method with a Crystal Screen (Hampton Research, Aliso Viejo, CA, USA) and Wizard Classic 1 and 2 (Rigaku Reagents, Bainbridge Island, WA, USA) at 293 K. The crystals of AtPsb27 were generated by mixing 1 µl protein with 1 µl reservoir solution containing 2.0 M potassium/sodium phosphate (pH 7.0), and crystals were observed after 3 days. The crystals were rapidly swept through 20% glycerol as cryoprotectant and were flash-cooled in liquid nitrogen. The X-ray crystallographic data were collected at the Beamline 17U1 at Shanghai Synchrotron Radiation Facility (SSRF), and data integration and scaling were completed with HKL2000. The structure was determined using molecular replacement program of ccp4 with TePsb27 (PDB code: 2Y6X) as the search model. The crystal structure model was then built manually and further refined with Coot, REFMAC5, and PHENIX. All the structural figures were prepared using PyMOL.

## Electronic supplementary material

Below is the link to the electronic supplementary material.

Supplementary material 1 (DOCX 2478 KB)
