# Supplementary material for: Crystal structure of Psb27 from Arabidopsis thaliana determined at a resolution of 1.85 Å
Source: Photosynth Res. 2017 Nov 2;136(2):139–46. doi: 10.1007/s11120-017-0450-3 (PMC5895690; doi:10.1007/s11120-017-0450-3)
Supplement: Supplementary file 1 — Supplementary material 1 (DOCX 2478 KB) [file 11120_2017_450_MOESM1_ESM.docx]

**Supplementary Materials**


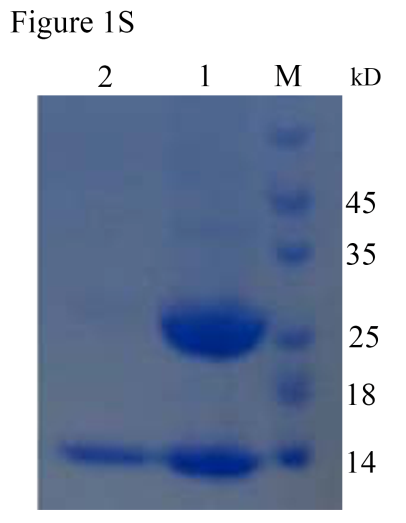


Figure 1S. Purification of mature Psb27 from Arabidopsis thaliana (AtPsb27).

Soluble protein samples were separated on a 15% (w/v) SDS-PAGE gel and stained with Coomassie blue.

Lane 1: GST-AtPsb27 was treated with TEV protease; lane 2: AtPsb27 protein after GST was removed by a GSH resin; lane M: prestained protein Marker (Fermentas, UK).


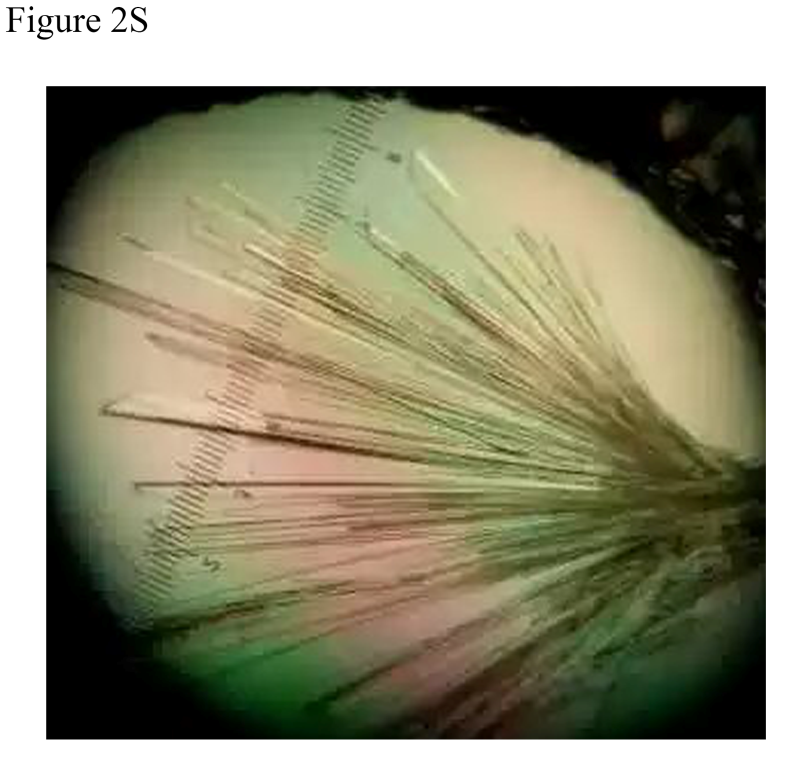


Figure 2S. Crystals of AtPsb27.

Crystals of AtPsb27 were obtained after a sequential step treatment of 1.6M Potassium/sodium phosphate pH 7.0, 2.0M Potassium/sodium phosphate pH 7.0, then 0.01M Sodium borate pH8.5/ 1.5M Sodium citrate tribasic dihydrate. The initial concentration of AtPsb27 is 22mg/ml. Crystals were formed after 3 days crystallization, and visualized with an optical microscope (CLS150X, Leica, UK)


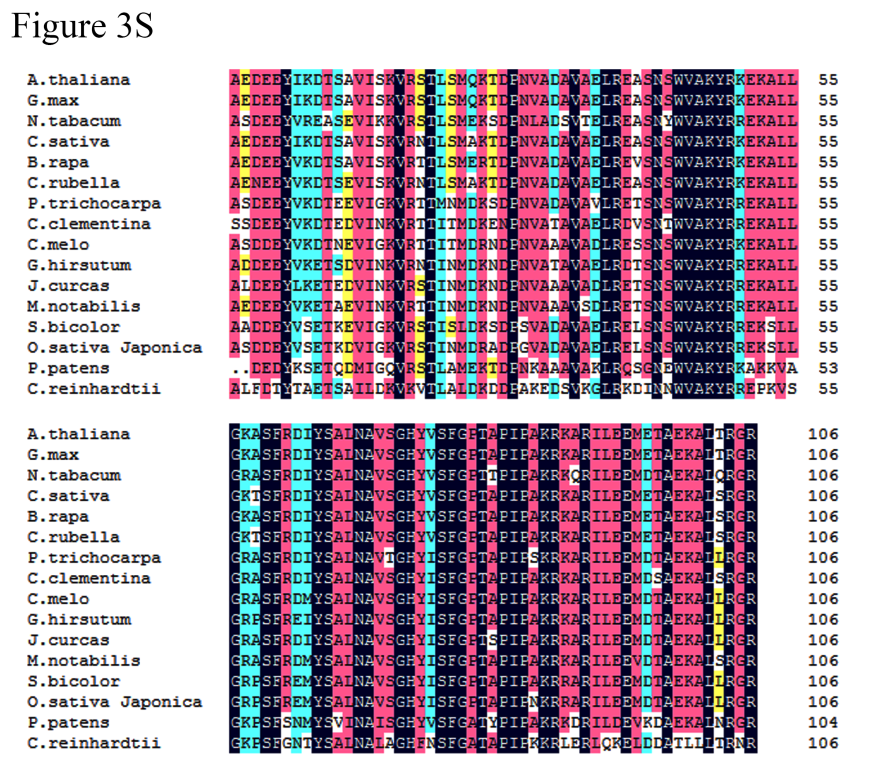


Figure 3S. Multiple amino acid sequence alignment of Psb27 proteins from land plants.

Psb27 mature protein sequences from 14 higher plants were aligned using ClustalX program, and Psb27 mature protein sequences from *Physcomitrella patens* and *Chlamydomonas reinhardtii* as two out-group sequences. The 14 species of land plants are *Arabidopsis thaliana, Glycine max, Nicotiana tabacum, Cannabis sativa, Brassica rapa, Capsella rubella, Populus trichocarpa, Citrus clementina, Cucumis melo, Gossypium hirsutum, Jatropha curcas, Morus notabilis, Sorghum bicolor, and Oryza sativa Japonica.*

Figure 4S


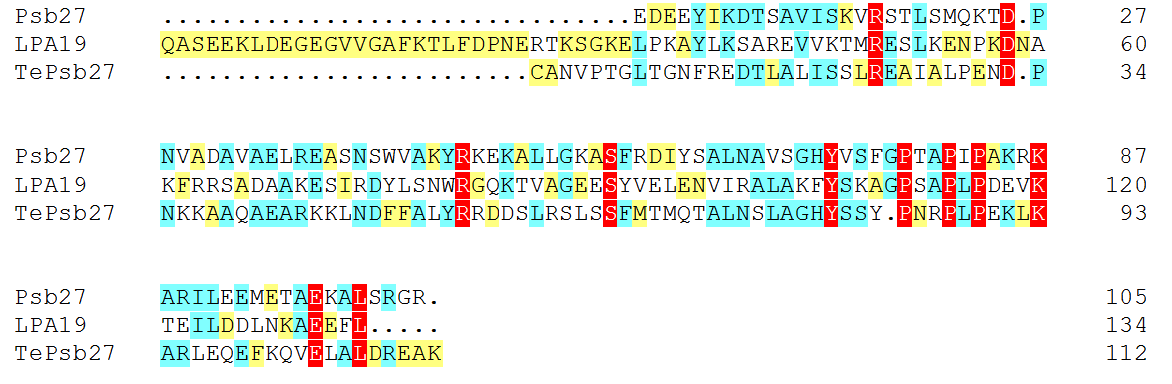


Figure 4S. Sequence alignment of Psb27, LPA19 from Arabidopsis and TePsb27

Mature protein sequences of Psb27 and LPA19 from Arabidopsis, and TePsb27 were aligned using ClustalX program.
